# The prevalence of alcohol-related deaths in autopsies performed in Lithuania between 2017 and 2020: a cross-sectional study

**DOI:** 10.1093/eurpub/ckae059

**Published:** 2024-03-28

**Authors:** Laura Miščikienė, Mindaugas Štelemėkas, Janina Petkevičienė, Jürgen Rehm, Shannon Lange, Justina Trišauskė

**Affiliations:** Health Research Institute, Faculty of Public Health, Lithuanian University of Health Sciences, Kaunas, Lithuania; Department of Health Management, Faculty of Public Health, Lithuanian University of Health Sciences, Kaunas, Lithuania; Health Research Institute, Faculty of Public Health, Lithuanian University of Health Sciences, Kaunas, Lithuania; Department of Preventive Medicine, Faculty of Public Health, Lithuanian University of Health Sciences, Kaunas, Lithuania; Health Research Institute, Faculty of Public Health, Lithuanian University of Health Sciences, Kaunas, Lithuania; Department of Preventive Medicine, Faculty of Public Health, Lithuanian University of Health Sciences, Kaunas, Lithuania; Centre for Addiction and Mental Health, Institute for Mental Health Policy Research, Toronto, ON, Canada; Centre for Addiction and Mental Health, Campbell Family Mental Health Research Institute, Toronto, ON, Canada; Department of Psychiatry, University of Toronto, Toronto, ON, Canada; Institute of Medical Science, University of Toronto, Toronto, ON, Canada; Dalla Lana School of Public Health, University of Toronto, Toronto, ON, Canada; Program on Substance Abuse & WHO European Region Collaboration Centre, Public Health Agency of Catalonia, Barcelona, Catalonia, Spain; Zentrum für Interdisziplinäre Suchtforschung, Universitätsklinikum Hamburg-Eppendorf, Hamburg, Germany; Centre for Addiction and Mental Health, Institute for Mental Health Policy Research, Toronto, ON, Canada; Centre for Addiction and Mental Health, Campbell Family Mental Health Research Institute, Toronto, ON, Canada; Department of Psychiatry, University of Toronto, Toronto, ON, Canada; Institute of Medical Science, University of Toronto, Toronto, ON, Canada; Health Research Institute, Faculty of Public Health, Lithuanian University of Health Sciences, Kaunas, Lithuania

## Abstract

**Background:**

Consumption of alcohol is a risk factor for non-communicable and infectious diseases, mental health problems, and can lead injuries and violence. The aim of this study was to evaluate the prevalence of alcohol-involved deaths among decedents who died of external causes and underwent autopsy in Lithuania.

**Methods:**

Study includes age persons of any age (from 0 to 110 years) who died and were autopsied in Lithuania from 1 January 2017 to 31 December 2020. Data were obtained from the Lithuanian State Register of Deaths and Their Causes.

**Results:**

Among external causes of death, the presence of alcohol was detected in 55.0% of cases. Male decedents had a significantly higher number of positive BAC level recorded, at 46.6%, compared with female decedents (32.1%; *P* < 0.001). The highest incidence of deaths where the alcohol was detected in the deceased’s blood was found when the decedent was listed as being in the victims of assault group (71.5%, 95% CI 65.4–77.2). However, the highest median BAC score was found for those in the accidents group (59.7%, 95% CI: 58.2–61.2, BAC 2.42 ‰, IQR 1.86).

**Conclusions:**

The findings of this study suggest that alcohol use may be a contributing factor in a wide range of fatal incidents, including accidents, injuries, and cases of violent intent. Inequalities between males and females were identified, with a higher proportion of males with alcohol detected in blood at the time of death.

## Introduction

Consumption of alcohol has a harmful effect on individual and population health. It is a risk factor for non-communicable and infectious diseases, mental health problems, and can lead to injuries and violence.^1^^,^^2^ The harmful use of alcohol resulted in 3 million deaths (5.3% of all deaths) worldwide in 2016. Of all deaths attributable to alcohol consumption worldwide, 28.7% were due to injuries, 21.3% due to digestive diseases, 19% due to cardiovascular diseases, 12.9% due to infectious diseases and 12.6% due to cancers.^3^

Alcohol affects various human cognitive functions, including divided attention, focused attention, and visuo-motor control,^4^ all of which increase the risk of ‘unintentional’ injuries. Unintentional injuries can be categorized as transport and non-transport depending on whether a vehicle of some kind was involved when the injury was sustained,^1^ and alcohol use is a risk factor for both non-transport and transport injuries.^1^ According to Lasota et al.,^5^ 72.2% of fatal pedestrian victims during the period of 2009–13 in Warsaw (Poland) were individuals under the influence of ethyl alcohol. Since alcohol use affects drivers’ attention, intoxicated passengers can distract drivers, and intoxicated pedestrians or cyclists can fall in the roadway, alcohol consumption is considered a risk factor for injuries for all road users.^6^^,^^7^ Other unintentional non-transport accidents are falls, drowning, poisoning, etc.^1^ Based on emergency room data from 28 countries, both frequent and heavy drinking was a predictor of injuries related to falls.^8^ In Sweden from 1992 to 2009, 38% of drowning victims tested for alcohol use had a positive blood alcohol concentration (BAC).^9^ Similar results were found in a meta-analysis—on an average 49.5% of fatal drownings involved the use of alcohol.^10^ Alcohol intoxication was also reported as common among incidents of accidental hypothermia.^11^

It is well known that ‘intentional’ injuries resulting from interpersonal violence and suicides can also be caused by persons under the influence of alcohol.^1^ For instance, it was found that individuals had an ∼7-fold increased risk for a suicide attempt soon after consuming alcohol, and this risk further increased to 37 times the average risk following the heavy use of alcohol (‘heavy drinking’ levels across studies varied from >100 g pure alcohol to BAC ≥ 0.10 mg/dl and 4+ drinks for females/5+ drinks for males)^12^ (underlying drinks here defined as 14 grs pure alcohol). It is not only drinking during the suicide event itself that increases risk, chronic heavy drinking is also linked to suicide risk. A meta-analysis found that individuals with an alcohol use disorder had a significantly higher risk of suicidal ideation, a suicide attempt, and death by suicide,^13^ compared with individuals without an alcohol use disorder.

Alcohol consumption also increases the risk of becoming a ‘victim’ of violence, assault.^14^ In the USA, 41.1% of homicide decedents had a positive BAC, and 27.7% had a BAC≥0.08 g/dl.^15^ Another study from the USA showed that over one third of firearm injury decedents (homicides and suicides) had consumed alcohol (any amount) prior to their deaths and that over one fourth of these had heavily consumed (blood–alcohol concentrations 0.08 g/dl or comparable amount indicated ‘heavy alcohol use’) alcohol prior to their deaths.^2^

Lithuania differs from other European countries in terms of both alcohol consumption habits and high mortality rates from external causes. Recorded alcohol consumption per person aged 15 and older was 11.2 l of absolute alcohol in 2022.^16^ However, consumption of alcohol in Lithuania has decreased in the past decade, and this decrease was one of the highest among OECD countries.^17^ The decrease in alcohol consumption in Lithuania and improvement in other related health indicators may be associated with the alcohol control policies implemented in the country, however Lithuania still remains among the countries with a high level of alcohol consumption.^17^^,^^18^ According to the Official Statistics Portal of Lithuania, deaths from external causes were recorded at 89.7 per 100 000 population, deaths from intentional self-harm (suicides) were recorded at 21.7 per 100 000 population, and deaths from transport crashes were recorded at 7.7 per 100 000 population in 2020.^16^ In the same year, fully alcohol-attributable mortality was reported at 21.8 per 100 000 population.^19^ Deaths in Lithuania decreased steadily from external causes over the 2000–20 period (from 145.8 per 100 000 population in 2000). Deaths from transport accidents have also been higher in the past, reaching 22.0 per 100 000 population in 2000. Intentional injuries rates were higher as well, for instance the suicide rate was 46.6 per 100 000 population. The assault and homicides rate declined from 9.9 per 100 000 in 2000–2.4 per 100 000 in 2020.^16^

For many years in Lithuania, when an autopsy examination was performed due to an external cause of death, a BAC score was not recorded in the mortality statistics. The State Register of Deaths and Their Causes (the Register) in Lithuania began operating only relatively recently, in 2010. The decision that information on concentrations of alcohol, medication and/or psychotropic substances was needed in this register was only made only in 2017, however.^20^^,^^21^ As a result of this decision, forensic data on the intoxication of deceased persons (including quantitative indicators of intoxication) were integrated into the register, and the State Forensic Medicine Service became the provider of this data. The State Forensic Medicine Service performs an autopsy for almost all deaths involving external causes (over 90%) and some deaths due to disease (∼16% of all deaths in Lithuania are investigated via autopsy).^22^

The aim of the current study was to evaluate the prevalence of alcohol-involved deaths in autopsied deaths in Lithuania between 2017 and 2020. We hypothesized that a greater proportion of decedents of external causes of death had consumed alcohol prior to their death, compared with those who died of other causes.

## Methods

This study includes persons of all ages (from 0 to 110 years) who died and were autopsied in Lithuania from 1 January 2017 to 31 December 2020. This period of time was selected due to the timing of the legal decision, as discussed above, to have information on the concentration of alcohol, medication and/or psychotropic substances included in the registry in 2017. Data were obtained from the Institute of Hygiene, which manages the register. The register contains data drawn from medical death and medical perinatal death certificates as well as other supporting documents.^23^

An autopsy is required by law under the following circumstances: a sudden or unexpected death, including those where violence is suspected; the cause of death is unclear; family members have requested one; cases where law enforcement is involved; deaths from injury, poisoning; and when the deceased is not identified. Further, if a person dies in an health care institution, a pathological examination is performed if there is demand of the family members or legal representatives, death was sudden or unexpected, the cause of death is unclear, if someone dies after surgical intervention, diagnostic, and treatment procedures, if someone dies (or is suspected to have died) from an occupational or infectious disease, a newborn or child (person under 18) dies, a pregnant woman or a woman who has recently given birth dies, a person dies who had been hospitalized for <24 h, and in other cases defined by law (as specified above). Upon the death of a person, a forensic medical examination, for purpose of pre-trial investigation shall be performed if the death occurs due to injury, poisoning or criminal abortion, the identity of the deceased is unknown, the cause of sudden death is suspected to be violence, the cause of death cannot be determined in other ways, and in other cases defined by law, upon the request of law enforcement authorities.^24^

The causes of death as recorded in the medical death certificate used in Lithuania corresponds to the World Health Organization (WHO) recommendations. Since 2011, the register uses the International Statistical Classification of Diseases and Related Health Problems 10th Revision (Australian Modification) (ICD-10-AM) for coding causes of death.^25^ The main (primary) cause of death is selected according to the ICD-10 coding rules set out in the methodological guidelines for classification (ICD-10).^23^

According to the register, in 2017, autopsies were performed in 14.9% of deaths, 14.9% in 2018, 14.4% in 2019, and 12.9% in 2020^23^^,^^26–28^ (number of cases provided in Supplementary table S1). However, in 18%, of these cases, alcohol was not evaluated (Supplementary table S2), and these cases were therefore not included in the data analysis of this study.

Deaths were analysed by sex, age, main cause of death, year of death, place of residence, and BAC level. The BAC was measured in permilles of on either volume or on grams (‰), depending on what was available for testing. The causes of death were divided into five main groups: accidents V00-X59, intentional self-harm (suicide) X60-X84, assault X85-Y09, event of undetermined intent Y10-Y34, and other causes. For a more detailed analysis, the causes of death were divided into 21 groups (tables 3–6). Analyses by year are provided in Supplementary tables S1–S3.

**Table 3:** Descriptive statistics and distribution (*n*, %) of positive alcohol cases by cause of death

Main cause of death	Total autopsies, *n*	Positive ethanol		Ethanol, median (min–max)	Interquartile range
		*n* (%)	CI		
**External causes V00-Y98**	**7550**	**4155 (55.0)**	**53.9–56.2**	**2.15 (0.15–8.74)**	**1.53**
**ACCIDENTS V00-X59**	4035	2410 (59.7)	58.2–61.2	2.42 (0.15–8.74)	1.86
*of which:*					
***Transport accidents* V00-V99**	*719*	*293 (40.8)*	*37.1–44.4*	*2.15 (0.15–8.74)*	*1.58*
Pedestrian injured in transport accident V00-V09	233	123 (52.8)	46.2–59.3	2.3 (0.16–3.98)	1.14
Pedal cyclist injured in transport accident V10-V19	41	23 (56.1)	39.7–71.5	2.15 (0.25–3.23)	1.56
Motorcyclist injured in transport accident V20-V39	58	9 (15.5)	7.3–20.7	2.16 (0.19–2.96)	1.30
Occupant of car injured in transport accident V40-V79	299	103 (34.4)	29.1–40.1	2.1 (0.15–8.74)	1.12
***Other accidents* W00-X59**	*3316*	*2117 (63.8)*	*62.2–65.5*	*2.48 (0.15–6.97)*	*1.64*
Falls W00-W19	431	172 (39.9)	35.3–44.7	2.09 (0.17–4.41)	1.46
Accidental drowning and submersion W65-W74	545	336 (61.7)	57.4–65.8	2.31 (0.15–4.43)	1.13
Effects of smoke, fire, and flame X00-X09	136	89 (65.4)	56.8–73.4	2.49 (0.16–4.18)	1.09
Effects of natural forces X30-X39	513	310 (60.4)	56.0–64.7	1.74 (0.16–5.13)	1.24
Exposure to excessive natural cold X31	512	310 (60.5)	56.2–64.8	1.74 (0.16–5.13)	1.24
Accidental poisoning by and exposure to noxious substances X40-X49	1083	868 (80.1)	77.6–82.5	3.16 (0.15–6.97)	1.91
Accidental poisoning by and exposure to narcotics X42	188	106 (56.4)	49.0–63.6	1.34 (0.16–3.68)	1.12
Accidental poisoning by and exposure to other and unspecified drugs, medicaments and biological substances X44	16	7 (43.8)	19.8–70.1	1.45 (0.25–3.08)	1.79
Accidental poisoning by and exposure to alcohol X45	601	569 (94.7)	92.6–96.3	3.64 (0.16–6.97)	1.17
**Intentional self-harm (suicides) X60-X84**	2556	1212 (47.4)	45.5–49.7	1.79 (0.15–8.28)	1.22
Intentional self-harm by hanging, strangulation and suffocation X70.0	2249	1100 (48.9)	46.8–51.0	1.78 (0.15–8.28)	1.19
**ASSAULT X85-Y09**	239	171 (71.5)	65.4–77.2	2.31 (0.18–4.08)	1.19
**Event of undetermined intent Y10-Y34**	716	360 (50.3)	46.6–54.0	1.95 (0.15–5.81)	1.76
**Other causes** *	11 326	3913 (34.5)	33.7–35.4	1.19 (0.1–7.76)	1.86
**Total**	18 872	8066 (42.7)	42.0–43.5	1.78 (0.10–8.74)	1.89

* Other causes—causes except V00-X59, X60-X84, X85-Y09, Y10-Y34.

**Table 4:** Distribution (*n*, %) of positive alcohol cases by cause of death and BAC group

Main cause of death	Total autopsies, n	BAC group, *n* (%)		
		0‰	0.1–0.79‰	≥0.8‰
**External causes V00-Y98**	**7550**	**3395 (45.0)**	**542 (7.2)**	**3613 (47.9)**
**ACCIDENTS V00-X59**	4035	1625 (40.3)	264 (6.5)	2146 (53.2)
*of which:*				
***Transport accidents* V00-V99**	*719*	*426 (59.2)*	*43 (6.0)*	*250 (34.8)*
Pedestrian injured in transport accident V00-V09	233	110 (47.2)	14 (6.0)	109 (46.8)
Pedal cyclist injured in transport accident V10-V19	41	18 (43.9)	4 (9.8)	19 (46.3)
Motorcyclist injured in transport accident V20-V39	58	49 (84.5)	1 (1.7)	8 (13.8)
Occupant of car injured in transport accident V40-V79	299	196 (65.6)	14 (4.7)	89 (29.8)
***Other accidents* W00-X59**	*3316*	*1199 (36.2)*	*221 (6.7)*	*1896 (57.2)*
Falls W00-W19	431	259 (60.1)	29 (6.7)	143 (33.2)
Accidental drowning and submersion W65-W74	545	209 (38.3)	29 (5.3)	307 (56.3)
Effects of smoke, fire, and flame X00-X09	136	47 (34.6)	4 (2.9)	85 (62.5)
Effects of natural forces X30-X39	513	203 (39.6)	48 (9.4)	262 (51.1)
Exposure to excessive natural cold X31	512	202 (39.5)	48 (9.4)	262 (51.2)
Accidental poisoning by and exposure to noxious substances X40-X49	1083	215 (19.9)	64 (5.9)	804 (74.2)
Accidental poisoning by and exposure to narcotics X42	188	82 (43.6)	24 (12.8)	82 (43.6)
Accidental poisoning by and exposure to other and unspecified drugs, medicaments and biological substances X44	16	9 (56.2)	2 (12.5)	5 (31.2)
Accidental poisoning by and exposure to alcohol X45	601	32 (5.3)	17 (2.8)	552 (91.8)
**Intentional self-harm (suicides) X60-X84**	2556	1344 (52.6)	196 (7.7)	1016 (39.7)
Intentional self-harm by hanging, strangulation and suffocation X70.0	2249	1149 (51.1)	172 (7.6)	928 (41.3)
**ASSAULT X85-Y09**	239	68 (28.5)	17 (7.1)	154 (64.4)
**Event of undetermined intent Y10-Y34**	716	356 (49.7)	65 (9.1)	295 (41.2)
**Other causes** *	11 326	7413 (65.4)	1504 (13.3)	2409 (21.3)
**Total**	18 872	10 806 (57.3)	2046 (10.8)	6020 (31.9)

* Other causes—causes except V00-X59, X60-X84, X85-Y09, Y10-Y34.

## Statistical methods

The Kolmogorov–Smirnov test was used to determine whether the distributions of quantitative data met the normality assumption. Abnormally distributed variables were described by median, minimal, maximal values, and interquartile range (IQR). The Mann–Whitney *U* test was used to compare two, and the Kruskal–Wallis to compare three and more independent samples. The categorical variables were presented as percentages, with confidence intervals (CIs), and compared using a chi-squared (χ^2^) test. Differences between groups in tables 1 and 2, and Supplementary table S5 were analysed using a *Z*-test with Bonferroni correction. Statistical significance was set at <0.05. All statistical analyses were performed using IBM SPSS, Version 27 for Windows.

**Table 1:** Distribution of positive autopsy cases by causes of death, demographic characteristics, season, death years, presence of alcohol in blood (*n*, %) and concentration of alcohol in blood (‰)

Characteristics	Total autopsies, *n*	Positive ethyl alcohol *n* (%)	Positive ethyl alcohol CI %	Median (min–max) (g/l)	Interquartile range
Total	18 872	8066 (42.7)	42.0–43.5	1.78 (0.10–8.74)	1.89
External causes V00-Y98	7550	4155 (55.0)	53.9–56.2	2.15 (0.15–8.74)	1.53
Other causes*	11 322	3911 (34.5)	33.7–35.4	1.19 (0.10–7.76)	1.86
*P*		<0.001		<0.001	
**Sex**					
Male	13 880	6464 (46.6)	45.7–47.4	1.81 (0.10–8.74)	1.86
Female	4992	1602 (32.1)	30.8–33.4	1.66 (0.14–6.97)	2.06
*P*		<0.001		0.003	
**Age group**					
0–19	293	46 (15.7)	11.7–20.4	1.09 (0.16–3.40)	1.29
20–34	1273	696 (54.7)	51.9–57.4	1.89 (0.15–6.62)	1.52
35–49	3409	1940 (56.9)	55.2–58.6	2.1 (0.15–8.28)	1.90
50–64	7089	3478 (49.1)	47.9–50.2	1.87 (0.1–8.74)	1.92
65 and more	6808	1906 (28.0)	26.6–29.1	1.29 (0.14–6.32)	1.83
*P*		<0.001		<0.001	
**Type of residence**					
City	11 758	4794 (40.8)	39.9–41.7	1.62 (0.1–6.97)	1.93
Rural	7114	3272 (46.0)	44.8–47.2	1.99 (0.15–8.74)	1.80
*P*		<0.001		<0.001	
**Years**					
2017	4915	2087 (42.5)	41.1–43.9	1.82 (0.14–8.28)	1.81
2018	4777	2042 (42.7)	41.3–44.2	1.79 (0.1–6.89)	1.83
2019	4484	1908 (42.6)	41.1–44.0	1.76 (0.14–6.97)	1.93
2020	4696	2029 (43.2)	41.8–44.6	1.75 (0.15–8.74)	2.01
*P*		0.887		0.366	
**Season**					
Spring (March–May)	4823	2115 (43.9)	42.4–45.3	1.81 (0.14–6.97)	1.88
Summer (June–August)	4387	1934 (44.1)^a^	42.6–45.6	1.73 (0.14–8.28)	1.93
Autumn (September–November)	4457	1836 (41.2)	39.7–42.7	1.78 (0.1–7.76)	1.90
Winter (December–February	5205	2181 (41.9)	40.6–43.3	1.82 (0.12–8.74)	1.87
*P*		0.009		0.005	

Blood alcohol concentration (BAC) refers to the percent of alcohol (ethyl alcohol or ethanol) in a person’s blood stream.

BAC by volume—1 permille (‰).

* Other causes—causes except V00-X59, X60-X84, X85-Y09, Y10-Y34.

a Difference between summer and autumn.

**Table 2:** Distribution of autopsy cases by causes of death, demographic characteristics, season, death years, presence of alcohol in blood (*n*, %) and concentration of alcohol in blood (‰)

Characteristics	Total autopsies, *n*	BAC group, *n* (%)		
		0‰	0.1–0.79‰	≥0.8‰
Total	18 872	10 806 (57.3)	2046 (10.8)	6020 (31.9)
External causes V00-Y98	7550	3395 (45.0)^a^	542 (7.2)^a^	3613 (47.9)^a^
Other causes*	11 322	7411 (65.5)	1504 (13.3)	2407 (21.3)
*P*		0.001		
**Sex**				
Male	13 880^b^	7416 (53.4)^b^	1588 (11.5)^b^	4876 (35.1)^b^
Female	4992	3390 (67.9)	458 (9.2)	1144 (22.9)
*P*		<0.001		
**Age group**				
0–19	293	247 (84.3)^c^	16 (5.5)^d^	30 (10.2)^e^
20–34	1273	577 (45.3)	120 (9.4)	576 (45.3)
35–49	3409	1469 (43.1)	380 (11.1)	1560 (45.8)
50–64	7089	3611 (50.9)	837 (11.8)	2641 (37.3)
65 and more	6808	4902 (72.0)	693 (10.2)	1213 (17.8)
*P*		<0.001		
**Type of residence**				
City	11 758^f^	6964 (59.2)^f^	1364 (11.6)^f^	3430 (29.2)^f^
Rural	7114	3842 (54.0)	682 (9.6)	2590 (36.4)
*P*		<0.001		
**Years**				
2017	4915	2828 (57.5)	492 (10.0)	1595 (32.5)
2018	4777	2735 (57.3)	504 (10.6)	1538 (32.2)
2019	4484	2576 (57.4)	503 (11.2)	1405 (31.3)
2020	4696	2667 (56.8)	547 (11.6)	1482 (31.6)
*P*		0.210		
**Season**				
Spring (March–May)	4823	2708 (56.1)	530 (11.0)	1585 (32.9)
Summer (June–August)	4387	2453 (55.9)^g^	553 (12.6)^g^	1381 (31.5)
Autumn (September–November)	4457	2621 (58.8)	464 (10.4)	1372 (30.8)
Winter (December–February	5205	3024 (58.1)	499 (9.6)	1682 (32.3)
*P*		<0.001		

Blood alcohol concentration (BAC) refers to the percent of alcohol (ethyl alcohol or ethanol) in a person’s blood stream.

BAC by volume—1 permille (‰).

* Other causes—causes except V00-X59, X60-X84, X85-Y09, Y10-Y34.

a Difference between external V00-Y98 and other causes.

b Difference between male and female.

c Difference between 0–19 and 20–34, 0–19 and 35–49, 0–19 and 50–64, 0–19 and 35 and more, 20–34 and 50–64, and 20–34 and 65 and more, 35–49 and 50–64, 35–49 and 65 and more, 50–64 and 65 and more;.

d Difference between 0–19 and 35–49, 0–19 and 50–64, 50–64 and 65 and more.

e Difference between 0–19 and 20–34, 0–19 and 35–49, 0–19 and 50–64, 0–19 and 35 and more, 20–34 and 50–64, and 20–34 and 65 and more, 35–49 and 50–64, 35–49 and 65 and more, 50–64 and 65 and more;.

f Difference between city and rural.

g Difference between summer and autumn, summer and winter.

## Results

Over the course of four years (2017–20), 18 872 autopsies were performed in Lithuania, and alcohol was detected in 42.7% of these cases (table 1). For cases of external causes of death, alcohol was detected in 55% of cases. A significantly higher proportion of male decedents had a positive BAC, at 46.6%, compared with female decedents (32.1%; *P* < 0.001). The highest proportion of deaths involving alcohol was found in those aged 20–49 years, where it was detected in over half the cases. A significantly higher number of deaths with a positive BAC was found among individuals living in a rural setting compared with those living in an urban setting (*P* < 0.001). There were no differences in the proportion of alcohol-positive cases between the years analysed and only one difference was noted between seasons (between summer and autumn) (table 1).

Among cases with a positive BAC, the BAC median was 1.78 (0.10–8.74) ‰ (table 1). The largest proportion (53.4% of male and 67.9% of female *P* < 0.001) had a BAC of 0‰, however, around one-third of the deceased had a BAC≥0.8 ‰ (table 2). Many significant differences between BAC groups were found for the different age groups, and some differences were noted between seasons (table 2). More detailed distributions of alcohol-positive cases and BAC scores of alcohol by sex and cause of death each year are presented in Supplementary table S3. Analyses by more specific BAC groups (0.1–0.39 ‰, 0.4–0.89 ‰, 0.9–1.39 ‰,1.4–1.99 ‰, 2.0–2.99 ‰, and 3.0 ‰ and more) are provided in Supplementary table S4.

The highest incidence of deaths where alcohol was detected in the deceased’s blood was found in individuals whose cause of death was categorized in the victims of assault group (71.5%, 95% CI 65.4–77.2). However, the highest median BAC level was found for the accidents group (59.7%, 95% CI: 58.2–61.2, BAC 2.42 ‰, IQR 1.86). Except for accidental poisoning by alcohol and exposure to alcohol, 94.7% (95% CI: 92.6–96.3, median 3.64‰, IQR 1.17). The highest BAC value was detected in cases in the group of individuals recorded as having died as a result of being an ‘occupant of car injured in transport accident V40-V79’—8.74 ‰ (table 3).

Among all cases of external causes of death, the largest proportion of cases had a BAC value ≥0.8 ‰ (47.9%). This was also the case for deaths resulting from accidents, cyclists injured in transport accidents, other accidents, accidental drownings and submersions, deaths due to the effects of smoke, fire, and flame, exposure to excessive natural cold, accidental poisoning by and exposure to noxious substances, accidental poisoning by and exposure to alcohol, and assault (table 4).

Alcohol was detected in 59.7% cases of accidents (table 3). Of these, 63.8% cases were men and 46.5% were women (Supplementary table S6). Among decedents of intentional self-harm (suicides; X60-X84), 52.6% of males and 26.4% of females had a positive BAC. The most commonly recorded type of intentional self-harm was reported as self-harm by hanging, strangulation, and suffocation. For this cause of death, alcohol was found in 54.1% of men and 26.7% of women. Alcohol was detected in half the cases in the undetermined intent category, in 55.4% of men and 36.1% of women. In the rest of the cases, alcohol-positive cases accounted for 34.5% (table 4), 37.1% of men, and 28.2% pf women (Supplementary table S6).

Significantly more male decedents had a positive BAC compared with female decedents for most of the deaths due to external causes. Among men and women, the highest BAC median was found when the cause of death was accidental poisoning by and exposure to alcohol, 3.64 (range 0.16–6.62) and 3.65 (range 0.16–6.97), respectively (Supplementary table S6). In males, a very high proportion of positive BAC scores (BAC≥0.8 ‰) were found among cases of death due to the effects of smoke, fire, and flame (X00-X09)a at 72.9%, accidental poisoning by and exposure to noxious substances (X40-X49) at 74.2%, accidental poisoning by and exposure to alcohol (X45) at 91%, and assault (X85-Y09) at 72.4%.

The female proportion of cases of external causes of death was lower compared with male. In females, the proportion of cases where the BAC was ≥0.8 ‰ was lower. Significant differences between male and female in BAC groups provided in Supplementary table S5.

## Discussion

The findings of this study emphasize the significant relationship between alcohol consumption and external causes of death based on Lithuanian autopsy data. The evidence presented suggests that alcohol use may be a contributing factor in a wide range of fatal incidents, including accidents, injuries, and cases of violent intent. We found that among external causes of death, alcohol was detected in 55% of deaths autopsied.

Results of this research showed inequalities between males and females, with a higher proportion of males having alcohol detected in blood at time of death. For instance, among cases of intentional self-harm (suicides), 52.6% of males autopsied had alcohol in their system, while alcohol was found in only 26.4% of females autopsied.

Our research corroborates the well-established link between alcohol use and an increased risk of accidents as the impairment of cognitive and motor skills under the influence of alcohol is a contributing factor to motor vehicle accidents, falls, and other unintentional injuries.^5^^,^^29^ In our study, in more than half the cases where the cause of death was recorded as an accident (59.7%), assault (71.5%), event of undetermined intent (50.3%), and about half of the cases of intentional self-harm (47.4%), alcohol was detected in the blood of decedents autopsied. In the context of violent deaths, our study also highlights the role of alcohol. We identified that alcohol was detected in 71.5% of assault-related deaths (i.e. victims). Kuhns et al.^14^ also determined the same link in their meta-analysis, but the proportion they found was 48%. Individuals under the influence of alcohol are more likely to engage in aggressive and violent behaviours, and this can increase the risk of homicides and assaults.^30^

However, Lithuanian police statistics show that perpetrators commit more than half of homicides when intoxicated by alcohol.^31^^,^^32^ Thus, violence and homicides seem to be caused by alcohol-fuelled situations where violence escalates, as both victim and aggressor are too intoxicated to find other solutions. This suggests that homicides may often happen during binge-drinking occasions and further analysis on the escalation of the dynamics involved is needed. High BAC levels have been consistently associated with a higher likelihood of being involved in accidents that result in fatal outcomes.^33^ Among all cases of external causes of death, around half (47.9%) happened when the BAC was 0.8 ‰ or higher.

When assessing external causes of death, alcohol concentration is not always recorded in mortality statistics, and this was the case in Lithuania for many years. The state forensic medicine agency did not always systematically collect this data for the analysis of alcohol influence. Until 2017, the data analysis was based on individual forensic experts. For instance, Benošis evaluated the association between external causes of deaths and alcohol (BAC) by analysing the Lithuanian forensic data for the period 1985–2014. Over those 29 years, 137 884 cases of external death were evaluated. A total of 77 718 deaths, or 56.5%, of those who died from external causes of death had consumed ethyl alcohol and were intoxicated at the time of their deaths.^34^

The objective data on level of alcohol intoxication serve for the international research community as an additional source of information allowing us to establish the extent of intoxication by alcohol in the deaths by external causes category in Lithuania. As Lithuania can be seen as an example of a natural experiment in the field of alcohol control,^18^ the now more thorough autopsy database, reviewed over a longer time period, will create opportunities to analyse the role and effects of alcohol in society, to assess the impact of alcohol control policies, as well to study the effects of the pandemic and post-pandemic periods on alcohol consumption in Lithuania.

The strengths of the study lie in the fact that due to current legislation, most (over 90%) deaths resulting from external causes undergo an autopsy, and this autopsy includes a requirement to record a BAC score. This study provides findings based on objectively measured indicators, unlike studies based on self-reported or the use of indirect data. However, it is important to acknowledge the limitations of this study. Our research focused on a specific time-period in one country, which may limit the generalizability of the findings. In some cases, when determining the cause of death, samples are not taken for alcohol testing (e.g. skeletonized corpses, burned, charred, long-term hospitalizations, etc.). The majority of autopsies is performed when there are external causes of death, and, thus, these data do not include other causes of death. We cannot determine causality of alcohol presence and cause of death by assessing immediate outcomes of fatalities associated with alcohol intake.

In conclusion, male decedents of external causes of death were more often intoxicated (i.e. had a BAC >0.8%) compared with their female counterparts. The prevalence of alcohol in deaths with performed autopsies in Lithuania is an important source of information in identifying the proportion of alcohol-attributable deaths in deaths resulting from external causes, and this study may serve as a base for future studies. The evidence presented highlights the importance of comprehensive efforts to address the role of alcohol in fatal incidents and ultimately to reduce the burden of these preventable tragedies on individuals and society as a whole.

## Supplementary Material

ckae059_Supplementary_Data

## Data Availability

The data underlying this article are available in the article and in its online supplementary material. Key points Among external causes of death, alcohol was detected in 55.0% of cases. A significantly higher proportion of male decedents had a positive BAC, compared with female decedents. Research provides insights into the role of alcohol in accidental deaths and intentional injuries and may serve as a base for future studies to identify any emerging patterns or changes.
