# Supplementary material for: The prevalence of alcohol-related deaths in autopsies performed in Lithuania between 2017 and 2020: a cross-sectional study
Source: Eur J Public Health. 2024 Mar 28;34(5):979–85. doi: 10.1093/eurpub/ckae059 (PMC11430968; doi:10.1093/eurpub/ckae059)
Supplement: ckae059_Supplementary_Data [file ckae059_supplementary_data.pdf]

Supplement 1.

Table 1. *Cases of death, autopsies and cases where alcohol was evaluated by year*

Year	Total cases of death (Register data)	Causes of autopsies (Register data)	Cases when alcohol was assessed (cases included in this study)
**2017**	40142	6001	4915
**2018**	39574	5895	4777
**2019**	38281	5515	4484
**2020**	43547	5605	4696

Table 2. *Causes of death by alcohol evaluation*

			Alcohol was evaluated		Alcohol was not evaluated		Total
W	Code	Cause of death (group)	Cases (n)	%	Cases (n)	%	n
1.	A00-B99	Certain infectious and parasitic diseases	94	51.4	89	48.6	183
2.	C00-D48	Neoplasms	247	40.6	361	59.4	608
3.	D50-D89	Diseases of the blood and blood-forming organs and certain disorders involving the immune mechanism	2	22.2	7	77.8	9
4.	E00-E90	Endocrine, nutritional and metabolic diseases	18	16.7	90	83.3	108
5.	F00-F99	Mental and behavioural disorders	13	52	12	48	25
6.	G00-G99	Diseases of the nervous system	84	65.6	44	34.4	128
7.	H00-H59	Diseases of the eye and adnexa	0	0	0	100	0
8.	H60-H95	Diseases of the ear and mastoid process	0	0	2	100	2
9.	I00-I99	Diseases of the circulatory system	8238	86.2	1318	13.8	9556
10.	J00-J99	Diseases of the respiratory system	634	80.6	153	19.4	787
11.	K00-K93	Diseases of the digestive system	1207	75.1	401	24.9	1608
12.	L00-L99	Diseases of the skin and subcutaneous tissue	1	12.5	7	87.5	8
13.	M00-M99	Diseases of the musculoskeletal system and connective tissue	1	6.7	14	93.3	15
14.	N00-N99	Diseases of the genitourinary system	33	47.8	36	52.2	69
15.	O00-O99	Pregnancy, childbirth and the puerperium	3	75	1	25	4
16.	P00-P96	Certain conditions originating in the perinatal period	6	8.1	68	91.9	74

17.	Q00-Q99	Congenital malformations, deformations and chromosomal abnormalities	11	16,7	55	83,3	66
18.	R00-R99	Symptoms, signs and abnormal clinical and laboratory findings, not elsewhere classified	701	59,4	479	40,6	1180
19.	S00-T98	Injury, poisoning and certain other consequences of external causes	0	0	0	100	0
20.	V01-Y98	External causes of morbidity and mortality	7550	88,4	992	11,6	8542
21.	Z00-Z99	Factors influencing health status and contact with health services	0	0	0	100	0
22.	U00-U85	Codes for special purposes	29	87,9	4	12,1	33
		Total	18872	82	4133	18	23005

Table 3. *Distribution (n, %) of cases by year of death, presence of alcohol in blood, sex and cause of death. Concentration of alcohol in blood (‰) among alcohol positive cases by sex, cause of death and year of death.*

**Years**	**2017**		**2018**		**2019**		**2020**	
	**Positive n (%)**	**BAC median, min max (‰)**	**Positive n (%)**	**BAC median, min max (‰)**	**Positive n (%)**	**BAC median, min max (‰)**	**Positive n (%)**	**BAC median, min max (‰)**
**Sex**								
Male	1686 (47.3)	1.85 (0.15-8.28)	1635 (46.5)	1.79 (0.10-6.29)	1544 (46.2)	1.80 (0.14-6.48)	1599 (46.2)	1.80 (0.15-8.74)
Female	401 (29.6)	1.64 (0.14-5.00)	407 (32.4)	1.78 (0.15-6.89)	364 (31.8)	1.69 (0.15-6.97)	430 (34.7)	1.52 (0.15-4.87)
**Pearson Chi-Square**	<0.001		<0.001		<0.001		<0.001	
**Age group**								
0-19	6 (8.5)	1.35 (0.16-3.09)	15 (19.7)	1.06 (0.21-2.92)	13 (18.6)	1.07 (0.18-2.28)	12 (15.8)	1.56 (0.26-3.40)
20-34	211 (57.3)	1.89 (0.16-5.37)	179 (53.3)	1.84 (0.16-5.32)	178 (57.4)	1.96 (0.15-5.56)	128 (49.4)	1.81 (0.16-6.62)
35-49	541 (57.4)	2.15 (0.15-8.28)	495 (56.8)	2.06 (0.15-6.89)	436 (58.1)	2.05 (0.15-6.97)	468 (55.3)	2.09 (0.15-6.42)
50-64	854 (48.0)	1.88 (0.15-7.22)	875 (50.0)	1.90 (0.10-6.29)	826 (47.2)	1.82 (0.14-6.31)	923 (51.0)	1.90 (0.15-8.74)
65 and more	475 (21.7)	1.38 (0.14-4.65)	478 (27.4)	1.27 (0.15-5.87)	455 (28.4)	1.33 (0.15-6.32)	498 (29.2)	1.17 (0.15-4.36)
**Main cause of death**								
Accidents V00-X59	699 (61.4)	2.36 (0.15-5.97)	612 (59.3)	2.42 (0.15-6.89)	546 (59.4)	2.45 (0.15-6.97)	553 (58.5)	2.44 (0.15-8.74)

Intentional self-harm (suicides) X60-X84	338 (47.8)	1.76 (0.15-8.28)	302 (46.5)	1.80 (0.15-4.42)	294 (47.0)	1.80 (0.16-5.28)	278 (48.3)	1.81 (0.16-4.26)
Assault X85-Y09	46 (68.7)	2.33 (0.19-4.08)	45 (76.3)	2.18 (0.21-3.95)	39 (72.2)	2.39 (0.18-3.80)	41 (69.5)	2.30 (0.19-4.00)
Event of undetermined intent Y10–Y34	88 (47.8)	1.88 (0.18-4.04)	101 (53.7)	2.05 (0.15-4.81)	86 (51.5)	2.02 (0.15-5.81)	85 (48.0)	1.85 (0.15-4.23)
Other causes *	916 (32.5)	1.28 (0.14-7.22)	982 (34.5)	1.18 (0.10-5.69)	943 (34.7)	1.13 (0.14-6.21)	1072 (36.5)	1.18 (0.15-7.76)
P	<0.001		<0.001		<0.001		<0.001	

* Other causes - causes except V00-X59, X60-X84, X85-Y09, Y10-Y34

Table 4. *Distribution (n, %) of cases by alcohol level in blood (‰) according to sex, age group, cause of death, and death year*

	Ethyl alcohol level (BAC, ‰) n (%)							
**2017-2020**	**0.1-0.39**	**0.4-0.89**	**0.9-1.39**	**1.4-1.99**	**2.0-2.99**	**3.0 and more**	**Total**	**p**
Total	1178 (14.6)	1076 (13.3)	970 (12.0)	1280 (15.9)	2163 (26.8)	1399 (17.4)	8066 (100)	
**Main cause of death**								
Accidents V00-X59	148 (6.1)	146 (6.0)	202 (8.4)	381 (15.8)	799 (33.2)	734 (30.5)	2410 (100)	<0.001
Intentional self-harm (suicides) X60-X84	103 (8.5)	126 (10.4)	188 (15.5)	295 (24.3)	421 (34.8)	79 (6.5)	1212 (100)	
Assault X85-Y09	13 (7.6)	4 (2.3)	13 (7.6)	27 (15.8)	81 (47.4)	33 (19.3)	171 (100)	
Event of undetermined intent Y10–Y34	37 (10.3)	39 (10.8)	42 (11.7)	69 (19.1)	100 (27.8)	73 (20.3)	360 (100)	
Other causes #	877 (22.4)	761 (19.4)	525 (13.4)	508 (13.0)	762 (19.5)	480 (12.3)	3913 (100)	
**Sex**								
Male	895 (13.9)*	849 (13.1)	781 (12.1)	1041 (16.1)	1787 (27.6)*	1111 (17.2)	6464 (100)	<0.001
Female	283 (17.6)	227 (14.2)	189 (11.8)	239 (14.9)	376 (23.5)	288 (18.0)	1602 (100)	
**Age group**								
0-19	9 (19.6)	9 (19.6)	10 (21.7)	9 (19.6)	5 (10.9)	4 (8.7)	46 (100)	<0.001
20-34	72 (10.3)	63 (9.1)	106 (15.2)	137 (19.7)	225 (32.3)	93 (13.4)	696 (100)	
35-49	193 (9.9)	231 (11.9)	194 (10.0)	300 (15.5)	565 (29.1)	457 (23.6)	1940 (100)	
50-64	472 (13.6)	451 (13.0)	403 (11.6)	547 (15.7)	958 (27.5)	647 (18.6)	3478 (100)	
65 and more	432 (22.7)	322 (16.9)	257 (13.5)	287 (15.1)	410 (21.5)	198 (10.4)	1906 (100)	
2017	289 (13.8)	249 (11.9)	268 (12.8)	334 (16.0)	580 (27.8)	367 (17.6)	2087 (100)	0.430
2018	282 (13.8)	271 (13.3)	246 (12.0)	341 (16.7)	556 (27.2)	346 (16.9)	2042 (100)	
2019	294 (15.4)	270 (14.2)	217 (11.4)	306 (16.0)	487 (25.5)	334 (17.5)	1908 (100)	

2020	313 (15.4)	286 (14.1)	239 (11.8)	299 (14.7)	540 (26.6)	352 (17.3)	2029 (100)	

[#] Other causes - causes except V00-X59, X60-X84, X85-Y09, Y10-Y34

* difference between male and female

Table 5. *Distribution (n, %) and descriptive statistics of positive alcohol cases by sex and cause of death*

	**Male**				**Female**			
	**Total**	**BAC group**			**Total**	**BAC group**		
**Main Cause of death**	**n**	**0 ‰**	**0.1-0.79 ‰**	**≥ 0.8 ‰**	**n**	**0 ‰**	**0.1-0.79 ‰**	**≥ 0.8 ‰**
**External causes V00-Y98**	**5832**	**2356 (40.4)**	**449 (7.7)**	**3027 (51.9)**	**1718**	**1039 (60.5)***	**93 (5.4) ***	**586 (34.1) ***
**ACCIDENTS V00-X59**	3076	1112 (36.2)	213 (6.9)	1715 (56.9)	959	513 (53.5) *	51 (5.3)	395 (41.2) *
of which:								
***Transport accidents* V00-V99**	530	282 (53.2)	34 (6.4)	214 (40.4)	189	144 (76.2) *	9 (4.8)	36 (19.0) *
Pedestrian injured in transport accident V00-V09	151	58 (38.4)	9 (6.0)	84 (55.6)	82	52 (63.4) *	5 (6.1)	25 (30.5) *
Pedal cyclist injured in transport accident V10-V19	36	14 (38.9)	4 (11.1)	18 (50.0)	5	4 (80.0)	0 (0.0)	1 (20.0)
Motorcyclist injured in transport accident V20-V39	55	46 (83.6)	1 (1.8)	8 (14.5)	3	3 (100)	0 (0.0)	0 (0.0)
Occupant of car injured in transport accident V40 -V79	207	118 (57.0)	10 (4.8)	79 (38.2)	92	78 (84.8) *	4 (4.3)	10 (10.9) *
Other accidents W00-X59	*2546*	*830 (32.6)*	*179 (7.0)*	*1537 (60.4)*	*770*	*369 (47.9) **	*42 (5.5)*	*359 (46.6) **
Falls W00-W19	311	165 (53.1)	27 (8.7)	119 (38.3)	120	94 (78.3) *	2 (1.7) *	24 (20.0) *
Accidental drowning and submersion W65-W74	425	136 (32.0)	23 (5.4)	266 (62.6)	120	73 (60.8) *	6 (5.0)	41 (34.2) *
Effects of smoke, fire, and flame X00-X09	96	24 (25.0)	2 (2.1)	70 (72.9)	40	23 (57.5) *	2 (5.0)	15 (37.5) *
Effects of natural forces X30-X39	379	138 (36.4)	39 (10.3)	202 (53.3)	134	65 (48.5) *	9 (6.7)	60 (44.8)
Exposure to excessive natural cold X31	379	138 (36.4)	39 (10.3)	202 (53.3)	133	64 (48.1) *	9 (6.8)	60 (45.1)
Accidental poisoning by and exposure to noxious substances X40-X49	856	167 (19.5)	64 (6.3)	635 (74.2)	227	48 (21.1)	10 (4.4)	169 (74.4)
Accidental poisoning by and exposure to narcotics X42	167	70 (41.9)	21 (12.6)	76 (45.5)	21	12 (57.1)	3 (14.3)	6 (28.6)
Accidental poisoning by and exposure to other and unspecified drugs, medicaments and biological substances X44	10	4 (40.0)	2 (20.0)	4 (40.0)	6	5 (83.3)	0 (0.0)	1 (16.7)
Accidental poisoning by and exposure to alcohol X45	467	28 (6.0)	14 (3.0)	425 (91.0)	134	4 (3.0)	3 (2.2)	127 (94.8)
**INTENTIONAL SELF-HARM (SUICIDES) X60-X84**	2048	970 (47.4)	172 (8.4)	906 (44.2)	508	374 (73.6) *	24 (4.7) *	110 (21.7) *
Intentional self-harm by hanging, strangulation and suffocation X70.0	1825	838 (45.9)	154 (8.4)	833 (45.6)	424	311 (73.3) *	18 (4.2) *	95 (22.4) *
**ASSAULT X85-Y09**	181	39 (21.5)	11 (6.1)	131 (72.4)	58	29 (50.0) *	6 (10.3)	23 (39.7) *
**EVENT OF UNDETERMINED INTENT Y10–Y34**	525	234 (44.6)	53 (10.1)	238 (45.3)	191	122 (63.9) *	12 (6.3)	57 (29.8) *

**OTHER CAUSES** **	8050	5061 (62.9)	1139 (14.1)	1850 (23.0)	3276	2352 (71.8) *	365 (11.1) *	559 (17.1) *
**Total**	13880 (73.5)	7416 (53.4)	1588 (11.5)	4876 (35.1)	4992 (26.5)	3390 (67.9) *	458 (9.2) *	1144 (22.9) *

* significant difference between men and women

** Other causes - causes except V00-X59, X60-X84, X85-Y09, Y10-Y34

Table 6. *Descriptive statistics and distribution (n, %) of positive alcohol cases by sex and cause of death*

	**Male**					**Female**					**p (between male and female)**
	**Total autopsies**	**Positive ethanol**				**Total autopsies**	**Positive ethanol**				
**Main Cause of death**	**n**	**Median ‰ (min - max)**	**Interquartile Range**	**n (%)**	**CI**	**n**	**Median ‰ (min - max)**	**Interquartile Range**	**n (%)**	**CI**	
**External causes V00-Y98**	**5832**	**2.15 (0.15-8.74)**	**1.51**	**3476 (43.3)**	**42.2-44.4**	**1718**	**2.16 (0.15-6.97)**	**1.61**	**679 (25.5) ***	**23.8-27.2**	<0.001
**ACCIDENTS V00-X59**	3076	2.43 (0.15-8.74)	1.57	1964 (63.8)	6.21-65.5	959	2.31 (0.15-6.97)	1.68	446 (46.5) *	43.3-49.7	<0.001
of which:											
***Transport accidents* V00-V99**	530	2.17 (0.15-8.74)	1.12	248 (46.8)	42.5-51.1	189	*1.91 (0.16-3.42)*	1.32	45 (23.8) *	*17.9-30.5*	<0.001
Pedestrian injured in transport accident V00-V09	151	2.54 (0.16-3.98)	1.00	93 (61.6)	53.3-69.4	82	1.94 (0.21-3.42)	1.21	30 (36.6) *	26.2-48.0	<0.001
Pedal cyclist injured in transport accident V10-V19	36	2.15 (0.25-3.23)	1.54	22 (61.1)	43.5-76.9	5	2.44 (2.44-2.44)	-	1 (20.0)	0.5-71.6	0.083
Motorcyclist injured in transport accident V20-V39	55	2.16 (0.19-2.96)	1.30	9 (16.4)	7.8-28.8	3	0	-	0 (0)	0	0.446
Occupant of car injured in transport accident V40 -V79	207	2.12 (0.15-8.74	1.01	89 (43.0)	36.2-50.0	92	1.89 (0.16-2.89)	1.85	14 (15.2) *	8.6-24.2	<0.001
***Other accidents* W00-X59**	*2546*	2.48 (0.15-6.62)	*1.62*	*1716 (67.4)*	*65.5-69.2*	*770*	*2.46 (0.15-6.97)*	*1.77*	*401 (52.1) **	*48.5-55.7*	<0.001
Falls W00-W19	311	2.09 (0.17-4.41)	1.53	146 (46.9)	41.3-52.7	120	2.17 (0.2-3.78)	1.42	26 (21.7) *	14.7-30.1	<0.001
Accidental drowning and submersion W65-W74	425	2.36 (0.15-4.43)	1.13	289 (68.0)	63.3-72.4	120	2.1 (0.15-3.59)	1.08	47 (39.2) *	30.4-48.5	<0.001
Effects of smoke, fire, and flame X00-X09	96	2.52 (0.16-4.18)	1.03	72 (75.0)	65.1-83.3	40	2.05 (0.18-3.60)	1.12	17 (42.5) *	27.0-59.1	<0.001
Effects of natural forces X30-X39	379	1.72 (0.16-5.13)	1.36	241 (63.6)	58.5-68.4	134	1.75 (0.2-3.04)	0.96	69 (51.5) *	42.7-60.2	0.014

Exposure to excessive natural cold X31	379	1.72 (0.16-5.13	1.34	241 (63.6)	58.5-68.4	133	1.75 (0.2-3.04)	0.96	69 (51.9) *	43.1-60.6	0.017
Accidental poisoning by and exposure to noxious substances X40-X49	856	3.09 (0.15-6.62)	1.94	689 (80.5)	77.7-83.1	227	3.35 (0.16-6.97)	1.53	179 (78.9)	73.0-84.0	0.583
Accidental poisoning by and exposure to narcotics X42	167	1.38 (0.16-3.68	1.15	97 (58.1)	50.2-65.7	21	1.17 (0.2-1.86)	1.15	9 (42.9)	21.8-66.0	0.185
Accidental poisoning by and exposure to other and unspecified drugs, medicaments and biological substances X44	10	1.24 (0.25-3.08)	2.05	6 (60.0)	26.2-87.8	6	1.45 (1.45-1.45)	-	1 (16.7)	0.4-64.1	0.091
Accidental poisoning by and exposure to alcohol X45	467	3.64 (0.16-6.62	1.20	439 (94.0)	91.5-96.0	134	3.65 (0.16-6.97)	1.14	130 (97.0)	92.5-99.2	0.171
**INTENTIONAL SELF-HARM (SUICIDES) X60-X84**	2048	1.8 (0.15-8.28)	1.21	1078 (52.6)	50.4-54.8	508	1.71 (0.18-3.11)	1.28	134 (26.4)*	22.6-30.4	<0.001
Intentional self-harm by hanging, strangulation and suffocation X70.0	1825	1.76 (0.15-8.28)	1.19	987 (54.1)	51.8-56.4	424	1.86 (0.18-3.11)	1.30	113 (26.7)*	22.5-31.1	0.001
**ASSAULT X85-Y09**	181	2.3 (0.19-4.0)	1.16	142 (78.5)	71.7-84.2	58	2.2 (0.18-4.08)	1.39	29 (50.0)*	36.6-63.4	<0.001
**EVENT OF UNDETERMINED INTENT Y10–Y34**	525	1.94 (0.15-4.57)	1.74	291 (55.4)	51.1-59.7	191	2.04 (0.15-5.81)	1.83	69 (36.1)*	29.3-43.4	<0.001
**OTHER CAUSES ****	8050	1.2 (0.1-7.76)	1.85	2989 (37.1)	36.1-38.2	3276	1.11 (0.14-5.05)	1.92	924 (28.2)*	26.7-29.8	<0.001
**Total**	13880 (73.5)	1.81 (0.10-8.74)	1.86	6464 (46.6)	45.7-47.4	4992 (26.5)	1.66 (0.14-6.97)	2.06	1602 (32.1)*	30.8-33.4	<0.001

* significant difference between men and women

** Other causes - causes except V00-X59, X60-X84, X85-Y09, Y10-Y34
